# Secondary data analysis of social care records to examine the provision of mental health support for young people in care

**DOI:** 10.1002/jcv2.12161

**Published:** 2023-03-28

**Authors:** Alice R. Phillips, Sarah L. Halligan, Megan Denne, Catherine Hamilton‐Giachritsis, John A. A. MacLeod, David Wilkins, Rachel M. Hiller

**Affiliations:** ^1^ Department of Psychology University of Bath Bath UK; ^2^ The National Institute for Health Research Applied Research Collaboration West (NIHR ARC West) University Hospitals Bristol and Weston NHS Foundation Trust Bath UK; ^3^ Centre for Academic Primary Care Bristol Medical School The University of Bristol Bristol UK; ^4^ Children's Social Care Research and Development Centre School of Social Sciences Cardiff University Cardiff UK; ^5^ Division of Psychology & Language Sciences University College London London UK; ^6^ Anna Freud Centre for Children and Families London UK

**Keywords:** maltreatment, mental health, social work, therapy, trauma

## Abstract

**Background:**

Young people in care are much more likely to experience mental health difficulties than the general population, yet little is known about the provision of mental health support for this group in the United Kingdom.

**Methods:**

Using routinely collected social care data, we explored the provision of mental health support for 112 young people in care in the UK. We identified young people experiencing elevated internalising or externalising difficulties in their first year in care (based on strengths and difficulties questionnaire scores) and extracted data on mental health referrals and provision. We generated descriptive statistics relating to provision of mental health support and used regressions to examine predictors of mental health provision, and associations between support and mental health outcomes one and 2 years later.

**Results:**

Eighty‐one percent of the children (*n* = 79) were referred to mental health services in their first year of being in care. Referrals were usually for emotional or conduct problems. Those with higher externalising symptoms were more likely to be referred than those with higher internalising symptoms (*OR* = 1.2, (95% confidence interval (CI): 1.01, 1.38)). Females were more likely to access support than males (*OR* = 3.82 (95% CI: 1.2, 13.3)). Sixty‐eight percent of children (*n* = 66) accessed mental health services in their first year of being in care. Of those who accessed services, support ended prematurely for 29 (44%) of them, often due to placement instability or disengagement. Accessing support in the first year of care was not associated with changes in mental health 1 year (*OR*: 2.14 (95% CI: 0.62,7.29)), or 2 years after entering care (*OR*: 0.72–8.57, (95% CI: 0.72, 8.57)), although methodological limitations are noted.

**Conclusions:**

Mental health difficulties for children in care are recognised quickly, but mental health support may be difficult to access, with issues evident in retention and engagement.

## Graphical abstract

We examined provision of mental health support for young people in care who had elevated mental health difficulties in their first year of being in care. Using secondary data analysis of anonymised social care records, we found that mental health difficulties were recognised and acted upon quickly with referrals, but that support was difficult to access with issues evident in retention and engagement. Access to support was unrelated to changes in mental health one and 2 years later, though concerns remain around the reliability of data.

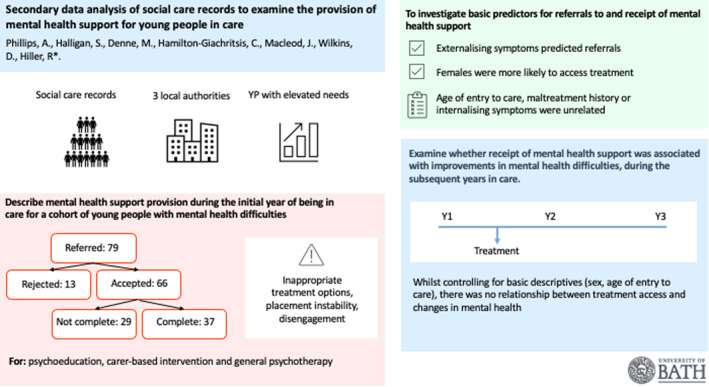

**Key Points**

- There are over 90,000 young people in care in the UK, and one in two meet the criteria for a diagnosable mental health condition.
- Drawing on routinely collected social care data, our findings show that where mental health difficulties are recognised, referrals are usually made relatively quickly (i.e., within the first year in care). However evidence‐based mental health support is difficult to access, with major issues evident around retention in support.
- Of particular relevance to health and social care policy, is that this work add weight to existing arguments around the need to close the need‐provision gap, which impacts a vulnerable group of young people.

## INTRODUCTION

There are currently 90,000 young people growing up in local authority care in the UK. Many have experienced significant adversity, including child maltreatment, often over many years (Solmi et al., 2021). Although entering care is a necessary and positive experience for some young people (Forrester et al., 2009), for others it is experienced as highly stressful, and it can entail further difficult experiences, such as the separation from siblings and frequent placement moves (Kothari et al., 2020). In the UK, the main reason for entering care is due to maltreatment or neglect, however there are a variety of legal pathways into care, and care placements (e.g., living with extended family members, foster carers or living in residential homes). Despite being a heterogeneous group, those in care are far more likely to meet the criteria for a mental health disorder than the general population, up to one in two children according to a recent meta‐analysis (Engler et al., 2022). Longitudinal research shows that removal from an adverse home environment into state care is not sufficient action to resolve their mental health difficulties, with problems often remaining fixed for several years (Hiller et al., 2022; Tarren‐Sweeney, 2017). Unaddressed mental health difficulties are considered a key driver for the disproportionate rate of challenging experiences in adulthood, such as homelessness and unemployment (Murray et al., 2020). Addressing this need may be one effective step towards mitigating some of the risk for adverse outcomes in adulthood.

In line with statutory guidance across the UK, the health and wellbeing of children in care is monitored annually, including assessment of mental health needs. The monitoring of this group during their initial years in care offers an opportunity to identify mental health problems early, allowing professionals to address the needs of young people quickly through the provision of mental health support (Arango et al., 2018). However, in the UK Child and Adolescent Mental Health Services (CAMHS) are oversubscribed and under‐resourced with long wait‐times and high rates of referral refusal (Birchwood & Singh, 2013; Frith, 2017). Although access can be difficult for many young people (McGorry et al., 2013), it is likely that care‐experienced people face unique challenges in accessing and engaging with professional mental health support. For example, there is some evidence that children in care are more likely to have referrals rejected than young people in the general population, though reasons for this remain unexplored (Hansen et al., 2021). Many young people in care experience frequent placement moves, meaning they do not have access to consistent advocates (e.g., teachers and foster parents) who can request mental health assessments or treatment, and navigate complex mental health services on their behalf (Beck, 2006). This is concerning, as we know that those with the most significant mental health difficulties are also most likely to be in unstable placements (Hiller et al., 2022). Aside from structural barriers to support, there is also evidence of some individual psychological barriers. For example, some care‐experienced people express ambivalence towards professional mental health interventions due to concerns around treatment options and apprehension about discussing historical negative experiences (Hiller et al., 2021; Powell et al., 2021). Given these known barriers, it is important that we examine whether we are adequately addressing the needs of young people in care, by examining the provision of mental health support.

Extant literature suggests that use of services is common amongst children in care, though this research is almost exclusively conducted in the United States, where mental health services function differently than those in the UK (e.g., McMillen et al., 2004). Much of this research is limited to self‐report survey data which broadly examines the “use” of mental health services, overlooking complexities with the entire help‐seeking journey, including referral refusal and premature treatment disengagement (Birchwood & Singh, 2013; Frith, 2017). The kind of support routinely offered to children in care in the UK is largely unknown, as is whether the support offered is effective at improving the mental health of young people in care. Understanding for whom accessing mental health support is most challenging is crucial to making services accessible and inclusive for those who require it.

Using secondary data analysis of routinely collected social care records, we aimed to: (1) describe mental health support provision during the initial year of being in care for a cohort of young people with mental health difficulties; (2) investigate basic predictors for referrals to and receipt of mental health support (sex, reports of neglect, total reports of abuse, age of removal into care, externalising and internalising symptoms); and (3) examine whether receipt of mental health support was associated with improvements in mental health difficulties, during the subsequent years in care.

## METHODS

### Procedure

The sample was obtained from an existing dataset collected by Hiller et al. (2022), which examined the mental health trajectories of young people during their initial years in care. Hiller et al. (2022) extracted information from electronic social care records from three local authorities in England. Local authorities provided completely anonymised data of all young people who entered care between 2012 and 2016, and who also remained in the care system for at least 2.5 years. In the current study, we have included only those young people where their carer‐reported strengths and difficulties questionnaire (SDQ; Goodman, 2001) scores placed them in the ‘abnormal’ range of difficulties within their first year in care. Ethical approval was obtained from the Bath Psychology Research Ethics Committee (REF: 16–284), with further approvals/permissions provided by participating local authorities.

### Data extraction and measures

#### Pre‐care descriptives

Basic descriptive information was extracted from social care files, and comprised of sex (male, female), ethnicity (white, black, mixed, and other), age at entry to care, and maltreatment history. Maltreatment history was gathered from chronologies, court reports, and police reports and coded as ‘present’ or ‘absent’ for sexual abuse, physical abuse, emotional abuse, experience of domestic abuse (i.e., witnessing), and neglect. A ‘total abuse’ variable was calculated by summing the presence of sexual abuse, physical abuse, emotional abuse and domestic abuse to create a continuous variable (0–4). Neglect maintained its categorical coding as ‘absent’ (0) or ‘present’ (1).

#### Mental health

Mental health was measured using the carer‐reported SDQ, a widely used and validated measure of social, emotional, and behavioural problems in 4–17‐year‐olds, which has also been validated for use with children in care (Goodman, 2001; Goodman & Goodman, 2012). The SDQ is collected annually by local authorities and returned to the government to monitor the mental health of all looked after children in England. Strengths and difficulties questionnaire data were collected for each participant across each of the first 3 years in care (see Hiller et al., 2022). Twenty‐items (five per subscale) measure internalising (two subscales: emotional problems, peer problems) and externalising (two subscales: attention problems, conduct problems) difficulties, with five additional items measuring pro‐social skills. Each item is rated on a 3‐point Likert scale from 0 (not true) to 2 (certainly true), resulting in subscale scores which range from 0 to 10, and a total problem score range of 0–40 where a higher score indicates greater difficulty (pro‐social skills are not included in the sum).

To identify participants who were experiencing mental health difficulties, the 3‐band categorisation system was used to divide the sample in to ‘normal’ (0–13), ‘borderline’ (14–16), and ‘abnormal’ (17–40) groupings (Goodman, 2001). As aforementioned, sample only includes young people who scored in the abnormal range during their first year in care.

To establish whether the mental health of participants reliably changed across Year 1 to Year 2, and Year 1 to Year 3, the Reliable Change Index (RCI; Jacobson & Truax, 1991) was calculated. Reliable Change Index can identify whether the score on a questionnaire has significantly changed using the standard deviation, whilst accounting for measurement error (Cronbach's Alpha; Jacobson & Truax, 1991). This means that RCI will indicate whether a change in SDQ score is significant and reliable (i.e., not due to measurement error), but cannot indicate whether the change is clinically relevant. In line with recommendations by Wolpert et al. (2015), rates of Crossing Clinical Threshold are also reported. Crossing Clinical Threshold calculates when an individual crosses between clinical and functional population scores (i.e., ‘abnormal’ to ‘borderline’, or ‘‘normal’). Crossing Clinical Threshold rates are presented for descriptive purposes only. In sum, for each participant we calculated whether their mental health reliably improved, and whether they had crossed clinical threshold across Y1‐Y2, and Y1‐Y3.

#### Mental health support provision

Mental health support provision ‘outcomes’ were gathered, which included: whether a referral was made to mental health services in the first year (yes, no) and whether the treatment was accessed (yes, no). Here, mental health services refer to all any primary or secondary health care service which supports the mental health of young people (e.g., CAMHS). This information was extracted from social care records, including clinical notes, outcome letters, referral and discharge letters, and the yearly health check. Accessing a treatment does not necessarily indicate that the young person completed treatment, but does demonstrate that they were at some point in receipt of professional mental health support, at least according to social care records. From the same files, we gathered information on the number of young people who experienced barriers for support at two points: (1) referral rejection, and (2) premature end to treatment. Where noted in files, provision ‘descriptives’ were gathered including: (i) reason for referral, (ii) the type of service referred to, (iii) the support they were recommended, (v) reason for referral rejection and (vi) reason for premature breakdown of therapy. Given the variety of sources used to gather this information, it was not always possible to identity who made treatment recommendations (e.g., social worker, mental health professional).

### Coding, data analyses and missing data

Coding manuals which were developed a priori were used to code the information obtained from social care records (e.g., reason for referral, reason for treatment breakdown etc.). The full definitions for how these categories were coded, and for the management of data where there were multiple referrals, is presented in Appendix S1. Codes were developed to closely represent raw data and did not involve subjective judgement; thus, we used quality checks rather than full inter‐rater reliability. Initial information was coded by trained junior researchers, before all data was checked by the first author. Any discrepancies were brought to a consensus meeting with the corresponding author.

To describe the sample, and provision of mental health support in the first year of entering care (research aim one), sample characteristics, support provision outcomes, and support provision descriptives were summarised using means and standard deviations for continuous data, and frequency and percentages for categorical data. We describe all data where it is available (i.e., regardless of ‘completeness’ of information around entire help‐seeking journey).

To examine basic predictors for referrals to and receipt of mental health support (research aim 2), tests for difference and correlations were performed to explore associations between relevant sample characteristics, and mental health during the first year in care with referrals to mental health support (yes/no), as well as access to treatment (yes/no). Externalising and internalising subscales of the SDQ were negatively correlated so they were examined as two continuous variables in terms of their relationship with mental health support provision (*r* = −0.4;*p* < 0.001). The sample had limited diversity in terms of ethnicity, so this data was excluded from analysis. Where associations were significant (*p* < 0.05) we used logistic regressions to explore the unique predictive value of variables. Children's social care records are notoriously inaccurate as a proxy of severity or complexity of maltreatment history (Asmussen et al., 2020). In this research, we use this information as an indicator of what might be known by social care staff about the child's maltreatment history. There were small percentages of data missing for referral and access to mental health support, so complete case analysis was conducted (Salgado et al., 2016).

To examine whether receipt of mental health support was associated with improvements in mental health difficulties during year 2 and year three of being in care (research aim 3), logistic regressions were planned. These examined whether there was a relationship between change in mental health across the initial years in care (reliable change/no reliable change in SDQ between Y1 to Y2, and Y1 to Y3), and access to mental health support (yes/no). We also included basic sex (male/female) and age of entering care (as a proxy of current age) as predictors of mental health outcomes. Due to missing data (i.e., Y2 or Y3 SDQs), we could calculate the RCI for 82 young people between Y1 and Y2 (*n* = 19 missing data), and 72 between Y1 and Y3 (*n* = 39 missing data). Chi‐squared tests revealed that there was no significant difference in number being referred to mental health services, or number accessing services, across those with and without SDQ missing data, therefore, we conducted complete case analysis.

## RESULTS

### Sample

The sample comprised of 112 participants who had abnormally elevated carer‐report SDQ scores in their first year in care. Three local authorities provided data: one covered urban areas and was medium sized (*n* = 48); another covered urban and rural areas and was medium sized (*n* = 52), and another covered urban areas and was smaller sized (*n* = 11). The average age of entry to care was 10.7 years old (SD = 3.6), ranging from 3.5 years old to 16 years old. Reflecting national statistics on young people in care, there were slightly more males than females (52% males, *n* = 58), and the sample was predominantly white (87%, *n* = 97; *n* = 4, 4% black; *n* = 6, 5% mixed ethnicity; and *n* = 4, 4% other). Reports of sexual abuse were found in 25% of records (*n* = 28), physical abuse in 62% (*n* = 69), emotional abuse in 70% (*n* = 78), experiencing domestic violence in 62% (*n* = 69) and neglect in 74% (*n* = 82). On average 2.2 (SD = 1.3) different types of maltreatment were mentioned in the records of young people.

### Mental health provision outcomes

Complete outcome data for the provision of mental health support were extracted for 97 young people who had abnormally elevated mental health scores (see Table 1). In sum, 19% of young people identified as struggling with their mental health did not receive a referral to mental health services in their first year of being in care (*n* = 18), 13% had a referral but this referral was rejected (*n* = 13), 30% accessed services but their support ended prematurely (*n* = 29), and for 38% of young people, there was evidence that they accessed to support and there was no information in the records to indicate that support ended prematurely (*n* = 37). Fifteen young people had partial or completely missing information on provision outcomes.

**TABLE 1:** Mental health provision outcomes.

Outcome	*n* (%)
	97^a^
Did not receive referral and they did not access support	18 (19%)
Received a referral but it was rejected	13 (13%)
Accessed support but it prematurely ended	29 (30%)
Accessed support and had no premature end to treatment	37 (38%)

^a^ total sample is *N* = 112, of which 97 had complete information about provision outcomes; *n* = 15 had partial or completely missing data.

The main reason given for referral rejection was that the treatment they were referred for was not recommended by the mental health professional (54%, *n* = 7). Other reasons included placement disruption or onwards referrals (46%, *n* = 6). Among the 29 premature treatment breakdowns, causes were identified as: placement instability (41%, *n* = 12): disengagement by the young person (17%, *n* = 5) or other disruptions (e.g., onwards referral, age out of services etc. 41%, *n* = 12).

### Mental health support descriptives

Descriptive referral data were extracted for 90 young people who received a referral in the first year of entering care (see Table 2). In sum, reasons for referral were diverse, with emotional problems (41%, *n* = 52), conduct problems (21%, *n* = 25), and risky behaviour (17%, *n* = 21) being the most common reported problems. The most common referral destination was specialist/targeted CAMHS (50%, *n* = 45). Recommended treatment was varied, but most often young people were recommended general psychotherapy (21%, *n* = 19) or psychoeducation (18%, *n* = 16).

**TABLE 2:** Mental health provision descriptives for young people who received a referral.

Descriptive	*N* (%)
	90^a^
Referral destination	
Specialist CAMHS	45 (50%)
CAMHS	14 (16%)
Specialist children in care service	13 (14%)
Specialist Non‐CAMHS	12 (13%)
School or education based treatment	4 (4%)
Insufficient information	2 (2%)
Recommended treatment^b^	
General psychotherapy	19 (21%)
Psychoeducation	16 (18%)
Carer or parent‐based support	12 (13%)
Play‐based or creative therapy	8 (9%)
Extended assessment	5 (6%)
Specialist intensive programme	4 (4%)
Psychopharmacology	3 (3%)
Cognitive‐based therapy	2 (2%)
Insufficient information	21 (23%)
Reason for referral^c^	
Emotional problems	52 (41%)
Conduct problems	25 (19%)
Risky behaviour	21 (16%)
External circumstances for referral	8 (6%)
Attachment problems	8 (6%)
Neurodevelopmental disorder	5 (4%)
Psychosis	1 (1%)
Insufficient information	9 (7%)

^a^ total sample *N* = 112, of which *n* = 90 had some descriptive information around provision of mental health support.

^b^ Recommendations taken from referral letters, or mental health assessments.

^c^ percentage of times this is mentioned (i.e., not percentage of young people).

### Predictors of mental health support provision

We used correlations (Kendall's tau b or point‐biserial correlation) and tests for difference (Fisher's Exact Test or Pearson's Chi‐Squared) to examine the associations between sex, reports of neglect, total reports of abuse, age of removal into care, externalising and internalising symptoms in the first year of being in care, and referrals (see Table 3). Analyses were conducted for young people which had both complete SDQ and information on whether a referral was made (*n* = 108). We found a small significant positive correlation between total externalising symptoms during the first year of being in care and being referred to mental health services $\left({n=90,\tau }_{b}=.20,p=.02\right)$. There was no evidence of associations between referrals and sex, maltreatment, age of entering care, or internalising symptoms. A logistic regression was performed to examine the predictive value of externalising symptoms, and the model was statistically significant (*χ* ^2^ (1) = 4.71, *ρ* = 0.003). Odds ratio indicated that a unit change in externalising symptoms was related to a 1.2 increase in the likelihood of being referred for support (OR: 1.2 (95% confidence interval (CI): 1,1.38); see Table 4).

**TABLE 3:** Cross‐tabulation of personal and care‐related characteristics and mental health service provision.

	Referral		Accessed support	
	Yes	No	Yes	No
*N* ^a^	90	18	66	17
Sex (*n*, %)				
Male	45 (79%)^b^	12 (21%)^b^	30 (70%)^c^*	13 (30%)^c^*
Female	45 (88%)^b^	6 (12%)^b^	36 (90%)^c^*	4 (10%)^c^*
Total abuse (M, SD)	2.2 (1.2)^d^	1.8 (1.2)^d^	2.1 (1)^d^	2.4 (1)^d^
Neglect (*n*, %)				
Reported	67 (84%)^b^	13 (16%)^b^	45 (74%)^c^*	16 (26%)^c^*
Not reported	23 (82%)^b^	5 (18%)^b^	21 (95%)^c^*	1 (5%)^c^*
Age of entry to care (M, SD)	11 (3.2)^d^	9.7 (4.2)^d^	10.5 (2.8)^d^	11.3 (3.6)^d^
Externalising symptoms (M, SD)	14.1 (3.2)^d^*	12.2 (3)^d^*	14 (3.1)^d^	14.7 (3.2)^d^
Internalising symptoms (M, SD)	11.2 (3.6)^e^	10.9 (2.9)^e^	11.4 (3.6)^c^	9.6 (3.5)^c^

^a^ total sample *N* = 112. Ns here are where we had complete information about provision of mental health support as well as SDQ data.

^b^ percentage of times this is mentioned (i.e., not percentage of young people).

^c^ Pearson's Chi‐Squared.

^d^ Kendall's tau b.

^e^ point‐biserial correlation.

*p < 0.05.

The same analyses were conducted to examine predictors for accessing support. Again, analyses were conducted for young people which had both complete SDQs and information on whether they accessed support (*n* = 83). Gender and reports of neglect were the only variables that were related to accessing support, whereby females were more likely to access support (*χ* ^2^ = 5.2, *ρ* = 0.03), as were those without reports of neglect (*χ* ^2^ = 4.7, *ρ* = 0.03). A logistic regression was performed to examine the unique predictive value of sex and presence/absence of neglect, and the model was statistically significant (*χ* ^2^ (2) = 10.8, *ρ* = 0.005; see Table 4). The model explained 19% of the variance in rates of accessing support. Once sex and neglect were entered into the model, sex was the only remaining significant predictor, whereby the odds ratio indicated that being female was related to a 3.8 times increase in likelihood of accessing support (*OR*:3.82 (95% CI: 1.2, 13.3)).

**TABLE 4:** Logistic regression examining predictive value of gender and provision of mental health support.

	β	SE	Wald	*df*	*p*	Odds ratio	95% Confidence interval
Referral to support							
Externalising symptoms	0.17	0.08	4.48	1	0.034	1.2	1.01–1.39
Constant	−0.64	1.06	0.36	1	0.55	0.53	
Access to support							
Sex (female)	1.34	0.64	4.42	1	0.035	3.82	1.2–13.3
Neglect (not reported)	−1.99	0.1.08	0.3.4	1	0.065	0.14	0.017–1.13
Constant	2.51	1.05	5.77	1	0.016	12.33	

### Mental health outcomes and mental health support provision

We examined whether accessing mental health support (yes/no) was related to changes in mental health across Y1 to Y2, and Y1 to Y3, using the RCI for the SDQ as the outcome. Around half of the sample reliably improved in terms of their SDQ scores between Y1‐Y2 (51% *n* = 42), with similar percentages between Y1‐Y3 (57%; *n* = 41). In most instances, reliable improvements by year two were maintained into year three, and most participants who had a reliable improvement (RCI), also had clinical ‘recovery’ (CCT; see Appendix S2 for details). There was also no evidence that sex and age of removal into care, were associated with change in scores, so these were not included as covariates in the logistic regression (see Table 5). Using a logistic regression, there was no evidence that accessing professional mental health support was associated with any change in mental health 1 year (*OR*: 2.13 (95% CI: 0.62, 7.29)) or 2 years later (*OR:* 2.48 (95% CI: 0.72, 8.57); see Table 6).

**TABLE 5:** Cross‐tabulation of pre‐care characteristics and reliable change in mental health.

	Year 1 to year 2		Year 1 to year 3	
	*n* = 62		*n* = 54	
	Reliable change	No reliable change	Reliable change	No reliable change
Sex (*n*, %)				
Male	21 (50%)^a^	21 (50%)^a^	23 (52%)^a^	21 (48%)^a^
Female	21 (52%)^a^	19 (48%)^a^	18 (64%)^a^	10 (36%)^a^
Age of entry to care (M, SD)	10.1 (3.4)^b^	10.8 (3.7)^b^	10.5 (3.5)^b^	10.3 (2.9)^b^
Access to support (*n*, %)				
Yes	26 (54%)^a^	22 (46%)^a^	26 (65%)^a^	14 (35%)^a^
No	5 (36%)^a^	9 (64%)^a^	6 (43%)^a^	8 (57%)^a^

^a^ Fisher's Exact Test.

^b^ Kendall's tau b.

**TABLE 6:** Logistic regression examining predictive value treatment access for reliable change in mental health.

	β	SE	Wald	*df*	*p*	Odds ratio	95% Confidence interval
Reliable change year 1‐year 2							
Access to support	0.76	0.63	1.44	1	0.23	2.13	0.62, 7.29
Constant	−0.59	0.56	1.11	1	0.292	0.56	
Reliable change year 1‐year 3							
Access to support	0.91	0.63	2.05	1	0.152	2.48	0.72, 8.57
Constant	−0.29	0.54	0.28	1	0.594	0.76	

Abbreviation: RCI, reliable change index.

## DISCUSSION

This project involved secondary data analysis of anonymised social care records for young people in care who were experiencing elevated emotional and/or behavioural difficulties in their first year of entering care, based on carer‐report SDQ. We sought to examine provision of mental health support and its influence upon mental health symptoms. We found high rates of referral to mental health services within the first year of entering care (81% of sample), though for almost 1 in 5 no referral was made. Issues arose following referral, whereby there were high rates of referral rejection, and support which broke down prematurely. We also found that those with greater externalising symptoms (but not internalising) were slightly more likely to be referred to mental health services, and that females were more likely to access mental health support than males. Analyses suggest that access to mental health treatment in the first year of being in care may not be associated with changes in mental health by year two and or three in care. Caution is warranted given concerns around data quality and the small sample size. Nevertheless, whether and how services address the mental health of young people in care remains an important area of future investigation.

Rates of referral to mental health services within the first year of entering care were high, but some were turned away from mental health services or and had treatment breakdowns (52% of the sample). Placement disruption, unsuitable treatment options and disengagement were common reasons for this. Placement instability is a well‐documented barrier to mental health treatment in this group (Beck, 2006), and is also associated with poorer mental health (Hiller et al., 2022). For this reason, it may be that those with the most significant mental health needs, who also have unstable placements, are also less likely to complete treatment. A synthesis of qualitative research also found that care‐experienced adults have reservations about engaging with mental health professionals (Powell et al., 2021), and this may be the case for young people whilst they are in care. Understanding how to make UK mental health services more accommodating for care‐experienced young people, and young people while they are in care, is a crucial but challenging area for research and services, as outlined in recent guidelines published by The National Institute for Health and Care Excellence (NICE, 2021). Accommodations could include safety nets which ensure the continuation of mental health care despite frequent changes in home addresses; capacity to allow children to re‐engage after a disengagement or non‐attendance easily and flexibly; or implementing programmes which address reservations and encourage the uptake and continued engagement in mental health support. Reviews accross the UK have identified a need to provide better mental health training to all professionals working with care‐experienced young people, and better strategic planning around mental health support (Independent Care Review, 2020; MacAlister, 2022).

In line with epidemiological research, emotional problems and conduct problems were the most common reasons for referral found in social care records (Ford et al., 2007). The treatments which were recommended were diverse, but given the limited information found within records, it is difficult to establish the type of support was delivered to the young people in practice (e.g., descriptions such as “general psychotherapy”). However, there is some evidence to suggest that children in care may have difficulties accessing evidence‐based mental health support for certain mental health conditions, and this requires further direct investigaiton (McGuire et al., 2022). What this also does raise is potential issues with record keeping within children's social care files. Particularly where a young person may not have a consistent adult to advocate for their needs, accurate record keeping and sharing is essential.

To understand whether certain groups of young people experience more challenges in receiving referrals, or accessing mental health services, we investigated associations between basic predictors (e.g., sex, age of entry to care), mental health (externalising and internalising symptoms) and referral to and access to mental health support. Largely, we were unable to identify basic predictors of access. Age of entry to care, extent of abuse, and the severity of internalising difficulties were not associated with referrals, or access to treatment. Higher levels of externalising symptoms were related to a small increase in the likelihood of being referred to mental health services, possibly due to the visibility of these problems by comparison to internalising difficulties. Females were almost four times more likely to have accessed mental health services. This may reflect higher refusal among young males, or poor availability of resources to address the mental health difficulties most commonly found in males (Fairchild et al., 2019).

Although half of the sample reliably improved in terms of their mental health between year 1 and year 2, and year 1 and year 3, half did not. Changes in mental health were not associated with access to mental health support. One reason for this may be the high rates of treatment breakdown (30% of the sample), or that young people in care can struggle to access evidence‐based treatments (McGuire et al., 2022). This may also reflect challenges with treating the mental health needs of children in care, given their complex mental health needs which could impact treatment response time (Lorenc et al., 2020). However, caution is warranted in drawing robust conclusions here. The sample size was limited and any number of other contextual factors are likely to impact mental health of young people over the first 3 years in care. For example, placement stability and placement type are all robustly associated with mental health outcomes (e.g., Engler et al., 2022). What these findings do show is a need for further focus on the mental health support available to young people in care, and whether it meets their needs. Of note, over half of the sample improved in terms of mental health regardless of their contact with mental health services. It is unclear within this research what lead to these positive changes, as several confounding factors, understood to impact mental health, were not included in analyses (e.g., placement stability, social support). Given limited resources within healthcare systems, it remains important to identify other ways in which we can meet the needs of young people in care beyond traditional mental health services. By focussing on building or restoring social support networks for example, (e.g., Holmes et al., 2020).

This research has many strengths, including the focus on a high‐needs yet under‐researched group, and the utilisation of existing data resources to develop basic information about access to mental health services that could inform practice and future research. However, findings should also be considered in light of limitations, which are primarily related to the reliance on social care data. Generally, social care records are kept for purposes of audit and inspection. It is in the interest of the social care organisation to keep records up to date, however the process by which information is collected and managed is unknown and systems are often hard to use (e.g., Hall et al., 2010). This means that the ‘completeness’ of information within social care records, and therefore its reliability is unknown ‐ a lack of information cannot be taken as evidence of absence. Similarly, it is not always clear whom is the source of information (e.g., social workers, mental health professionals). This research highlights how the importance of accurate records at the social care level, including from mental health services. Secondly, data was drawn from three local authorities and therefore findings relate to their specific service pathways. Some regions do not have specialised or targeted services for example, limiting generalisability. The sample size is also small, limiting the conclusions which can be drawn from analyses. Large‐scale (UK‐wide) research must be conducted to further our understanding of how we are serving the mental health needs of this vulnerable population, perhaps triangulating social care with healthcare records given concerns around the reliability of administrative data sources. Finally, this research investigates provision of mental health support for young people who enter care with highly elevated scores on the SDQ. We do not have information on help‐seeking activities undergone before being placed in care (e.g., referrals). The SDQ has been shown to be a good tool to indicate the presence of mental health disorders in children in care (Goodman et al., 2004). However, concern has also been raised at the ability for the SDQ to detect issues such as attachment difficulties, and that the thresholds from the SDQ may be too high for children in care (Wright et al., 2019). If this were the case, our use of the ‘abnormal’ range may only reflect an inclusion of only those specific difficulties, and thus may not be generalisable to those with more moderate difficulties.

## CONCLUSION

By conducting secondary data analysis of the social care records for young people in their first year of care who also have elevated mental health needs, we found that rates of referral to mental health support were high, but issues with accessing services arose following referral. Fifty‐two percent of the time referrals were rejected, or treatment ended prematurely. Analyses revealed that accessing services was unrelated to reliable improvement in mental health problems 1 year and 2 years after entering care, though limitations exist around the reliability of data. This work highlights a need‐provision disparity which impacts a highly vulnerable group of young people. The mental health needs of children in care need to be adequately supported as early as possible to avert the disproportionate rate of poor outcomes in adulthood for this group.

## AUTHOR CONTRIBUTIONS

**Alice Phillips**: Conceptualization; Data curation; Formal analysis; Project administration; Writing – original draft; Writing – review & editing. **Sarah Halligan**: Conceptualization; Methodology; Supervision; Writing – review & editing. **Megan Denne**: Data curation. **Catherine Hamilton‐Giachritsis**: Supervision; Writing – review & editing. **John Macleod**: Supervision; Writing – review & editing. **David Wilkins**: Supervision; Writing – review & editing. **Rachel Hiller**: Conceptualization; Funding acquisition; Supervision; Writing – review & editing.

## CONFLICTS OF INTEREST STATEMENT

The authors have declared they have no competing or potential conflicts of interest.

## Ethical Consideration

Ethical approval was obtained from the University of Bath Psychology Research Ethics Committee (study number: 16–284), with further approvals/permissions provided by participating local authorities. This is secondary data analysis project using routinely collected data from social care records. As a service evaluation project which stands to significantly benefit the service, access to service data was possible without participant consent. Of note, no identifiable data was extracted from the service database.

## Supporting information

Supplementary Information S1

## Data Availability

Research data are not shared.

## References

[jcv212161-bib-0001] Arango, C. , Díaz‐Caneja, C. M. , McGorry, P. D. , Rapoport, J. , Sommer, I. E. , Vorstman, J. A. , McDaid, D. , Marín, O. , Serrano‐Drozdowskyj, E. , Freedman, R. , & Carpenter, W. (2018). Preventive strategies for mental health. The Lancet Psychiatry, 5(7), 591–604. 10.1016/S2215-0366(18)30057-9 29773478

[jcv212161-bib-0002] Asmussen, K. , Fischer, F. , Drayton, E. , & McBribe, T. (2020). Adverse Childhood Experiences: What we know, what we don’t know, and what should happen next.

[jcv212161-bib-0003] Beck, A. (2006). Addressing the mental health needs of looked after children who move placement frequently. Adoption and Fostering, 30(3), 60–65. 10.1177/030857590603000308

[jcv212161-bib-0004] Birchwood, M. , & Singh, S. P. (2013). Mental health services for young people: Matching the service to the need. British Journal of Psychiatry, 202(s54), s1–s2. 10.1192/bjp.bp.112.119149 23288494

[jcv212161-bib-0005] Engler, A. D. , Sarpong, K. O. , Van Horne, B. S. , Greeley, C. S. , & Keefe, R. J. (2022). A systematic review of mental health disorders of children in foster care. Trauma, Violence, & Abuse, 23(1), 255–264. 10.1177/1524838020941197 32686611

[jcv212161-bib-0006] Fairchild, G. , Hawes, D. J. , Frick, P. J. , Copeland, W. E. , Odgers, C. L. , Franke, B. , Freitag, C. M. , & De Brito, S. A. (2019). Conduct disorder. Nature Reviews Disease Primers, 5(1), 43. 10.1038/s41572-019-0095-y 31249310

[jcv212161-bib-0007] Ford, T. , Vostanis, P. , Meltzer, H. , & Goodman, R. (2007). Psychiatric disorder among British children looked after by local authorities: Comparison with children living in private households. British Journal of Psychiatry, 190(4), 319–325. 10.1192/bjp.bp.106.025023 17401038

[jcv212161-bib-0008] Forrester, D. , Goodman, K. , Cocker, C. , Binnie, C. , & Jensch, G. (2009). What is the impact of public care on children's welfare? A review of research findings from England and wales and their policy implications. Journal of Social Policy, 38(3), 439–456. 10.1017/S0047279409003110

[jcv212161-bib-0009] Frith, E. (2017). Access and waiting times in children and young people’s mental health services. Education Policy Institute.

[jcv212161-bib-0010] Goodman, A. , & Goodman, R. (2012). Strengths and Difficulties Questionnaire scores and mental health in looked after children. The British Journal of Psychiatry : The Journal of Mental Science, 200(5), 426–427. 10.1192/bjp.bp.111.104380 22550331

[jcv212161-bib-0011] Goodman, R. (2001). Psychometric properties of the strengths and difficulties questionnaire. Journal of the American Academy of Child & Adolescent Psychiatry, 40(11), 1337–1345. 10.1097/00004583-200111000-00015 11699809

[jcv212161-bib-0012] Goodman, R. , Ford, T. , Corbin, T. , & Meltzer, H. (2004). Using the Strengths and Difficulties Questionnaire (SDQ) multi‐informant algorithm to screen looked‐after children for psychiatric disorders. European Child & Adolescent Psychiatry, 13(Suppl 2), Ii25–31. 10.1007/s00787-004-2005-3 15243783

[jcv212161-bib-0013] Hall, C. , Parton, N. , Peckover, S. , & White, S. (2010). Child‐centric information and communication technology (ICT) and the fragmentation of child welfare practice in England. Journal of Social Policy, 39(3), 393–413. 10.1017/S0047279410000012

[jcv212161-bib-0014] Hansen, A. S. , Christoffersen, C. H. , Telléus, G. K. , & Lauritsen, M. B. (2021). Referral patterns to outpatient child and adolescent mental health services and factors associated with referrals being rejected. A cross‐sectional observational study. BMC Health Services Research, 21(1), 1063. 10.1186/s12913-021-07114-8 34625073 PMC8501731

[jcv212161-bib-0015] Hiller, R. M. , Fraser, A. , Denne, M. , Bauer, A. , & Halligan, S. L. (2022). The development of young peoples’ internalising and externalising difficulties over the first three‐years in the public care system. Child Maltreatment, 0(0), 1–11. 10.1177/10775595211070765 PMC9716486 35081783

[jcv212161-bib-0016] Hiller, R. M. , Halligan, S. L. , Meiser‐Stedman, R. , Elliott, E. , Rutter‐Eley, E. , & Hutt, T. (2021). Coping and support‐seeking in out‐of‐home care: A qualitative study of the views of young people in care in England. BMJ Open, 11(2), e038461. 10.1136/bmjopen-2020-038461 PMC7887338 33589445

[jcv212161-bib-0017] Holmes, L. , Neagu, M. , Sanders‐Ellis, D. , & Harrison, N. (2020). Lifelong links: Evaluation report. https://assets.publishing.service.gov.uk/government/uploads/system/uploads/attachment_data/file/955953/Lifelong_Links_evaluation_report.pdf

[jcv212161-bib-0018] Independent Care Review . (2020). The promise. Retrieved from: https://www.carereview.scot/wp‐content/uploads/2020/02/The‐Promise.pdf

[jcv212161-bib-0019] Jacobson, N. S. , & Truax, P. (1991). Clinical significance: A statistical approach to defining meaningful change in psychotherapy research. Journal of Consulting and Clinical Psychology, 59(1), 12–19. 10.1037//0022-006x.59.1.12 2002127

[jcv212161-bib-0020] Kothari, B. H. , Blakeslee, J. , & Miller, R. (2020) Individual and interpersonal factors associated with psychosocial functioning among adolescents in foster care: A scoping review. Children and Youth Services Review. 118, 105454. 10.1016/j.childyouth.2020.105454 34887607 PMC8653982

[jcv212161-bib-0021] Lorenc, T. , Lester, S. , Sutcliffe, K. , Stansfield, C. , & Thomas, J. (2020). Interventions to support people exposed to adverse childhood experiences: Systematic review of systematic reviews. BMC Public Health, 20(1), 657. 10.1186/s12889-020-08789-0 32397975 PMC7216383

[jcv212161-bib-0022] MacAlister , J. (2022) The independent review of children's social care: Final report. The independent review of children's social care.

[jcv212161-bib-0023] McGorry, P. , Bates, T. , & Birchwood, M. (2013). Designing youth mental health services for the 21st century: Examples from Australia, Ireland and the UK. British Journal of Psychiatry, 202(s54), s30–s35. 10.1192/bjp.bp.112.119214 23288499

[jcv212161-bib-0024] McGuire, R. , Halligan, S. L. , Meiser‐Stedman, R. , Durbin, L. , & Hiller, R. M. (2022). Differences in the diagnosis and treatment decisions for children in care compared to their peers: An experimental study on post‐traumatic stress disorder. British Journal of Clinical Psychology, 00(4), 1–14. 10.1111/bjc.12379 PMC9796033 35702815

[jcv212161-bib-0025] McMillen, J. C. , Scott, L. D. , Zima, B. T. , Ollie, M. T. , Munson, M. R. , & Spitznagel, E. (2004). Use of mental health services among older youths in foster care. Psychiatric Services, 55(7), 811–817. 10.1176/appi.ps.55.7.811 15232022

[jcv212161-bib-0026] Murray, E. T. , Lacey, R. , Maughan, B. , & Sacker, A. (2020). Association of childhood out‐of‐home care status with all‐cause mortality up to 42‐years later: Office of National Statistics Longitudinal Study. BMC Public Health, 20(1), 735. 10.1186/s12889-020-08867-3 32434479 PMC7238620

[jcv212161-bib-0027] National Insitute for Health and Care Excellence . (2021). Looked after children and young people. Retrieved from https://www.nice.org.uk/guidance/ng205 34941234

[jcv212161-bib-0028] Powell, K. , Huxley, E. , & Townsend, M. L. (2021). Mental health help seeking in young people and carers in out of home care: A systematic review. Children and Youth Services Review. 127, 106088. 10.1016/j.childyouth.2021.106088

[jcv212161-bib-0029] Salgado, C. M. , Azevedo, C. , Proença, H. , & Vieira, S. M. (2016). Chatper 13: Missing data. In Secondary analysis of electronic health records (1st ed, 143–161). Springer International Publishing: Imprint: Springer.

[jcv212161-bib-0030] Solmi, M. , Radua, J. , Olivola, M. , Croce, E. , Soardo, L. , Salazar de Pablo, G. , Il Shin, J. , Kirkbride, J. B. , Jones, P. , Kim, J. H. , Kim, J. Y. , Carvalho, A. F. , Seeman, M. V. , Correll, C. U. , & Fusar‐Poli, P. (2021). Age at onset of mental disorders worldwide: Large‐scale meta‐analysis of 192 epidemiological studies. Molecular Psychiatry, 27(1), 281–295. 10.1038/s41380-021-01161-7 34079068 PMC8960395

[jcv212161-bib-0031] Tarren‐Sweeney, M. (2017). Rates of meaningful change in the mental health of children in long‐term out‐of‐home care: A seven‐to nine‐year prospective study. Child Abuse & Neglect. 72, 1–9. 10.1016/j.chiabu.2017.07.002 28734200

[jcv212161-bib-0032] Wolpert, M. , Görzig, A. , Deighton, J. , Fugard, A. J. B. , Newman, R. , & Ford, T. (2015). Comparison of indices of clinically meaningful change in child and adolescent mental health services: Difference scores, reliable change, crossing clinical thresholds and ‘added value’ – an exploration using parent rated scores on the SDQ. Child and Adolescent Mental Health. 20(2), 94–101. 10.1111/camh.12080 32680384

[jcv212161-bib-0033] Wright, H. , Wellsted, D. , Gratton, J. , Besser, S. J. , & Midgley, N. (2019). Use of the Strengths and Difficulties Questionnaire to identify treatment needs in looked‐after children referred to CAMHS. Developmental Child Welfare, 1(2), 159–176. 10.1177/2516103218817555

