# Supplementary material for: Secondary data analysis of social care records to examine the provision of mental health support for young people in care
Source: JCPP Adv. 2023 Mar 28;3(2):e12161. doi: 10.1002/jcv2.12161 (PMC10519729; doi:10.1002/jcv2.12161)
Supplement: Supplementary file 1 — Supplementary Information S1 [file JCV2-3-e12161-s001.docx]

**Supporting Information**

**Appendix S1.**

**Coding reason for referrals**

‘Reason for referral’ represents the problems that were given in referral or assessment letters to describe the difficulties the young person was facing. Where there was sufficient information, descriptions were split into several categories, including: emotional problems (e.g., anxiety, low mood); conduct problems; risky behaviour (e.g., alcohol misuse, risky sexual behaviour); attachment problems; neurodevelopmental disorders; psychosis and finally, external circumstances for referral (e.g., placement instability). These categories covered all of the reasons reported in the available data. Most of the time, just one reason for referral was mentioned, come records described more than one problem. To demonstrate the ubiquity of different psychological difficulties, the descriptives given are the number and percentages of records which mention each psychological problem. This means that those young people with more than one ‘reason for referral’, will appear in more than one problem category (e.g., a young person with attachment problems as well as risky behaviour will appear in the counts of both).

**Coding referral destination**

‘Referral destination’ is the type service that young people were referred to, based on referral letters. This was split into five categories: CAMHS; CAMHS Specialist support (specialised support for specific groups or mental health problems (e.g., substance misuse)); specialist non-CAMHS (specialised support for mental health problems but are not part of CAMHS (e.g., charity sector organisations)); Non-NHS specialist children in care services (therapeutic support for children in care or their families, often through social services); and finally, education and school based support (e.g., referral to specialist school).

Oftentimes, assessment and referral letters gave recommendations for the type of support which might be useful for the young person. Where this information was gathered from referral letters, it is likely their GP or social worker provided recommendations, and where this came from assessment letters it is likely a clinical psychologist provided the recommendation. We categorised recommended treatment into eight broad categories related to support type, including: psychoeducation, unspecified psychotherapy, carer or parent-based support, play-based of creative therapy (e.g., art or drama therapy), extended assessment of needs, psychopharmacology, specialist intensive programmes and cognitive-behavioural style therapy. See below for handling of cases where multiple treatment recommendations were made within a referral.

We were also able to gather reasons for referral rejection, and reasons for breakdown from records. These were split into 7 categories: (i) treatment not recommended (e.g., unsuitable treatment options), (ii) young person did not engage, (iii) family or carer did not engage, (iv) placement disruption, (v) other external disruption (e.g., ongoing court proceedings), (vi) onwards referral to another service and (vii) transition to adult services.

**Managing multiple referrals**

We managed to extract information about referrals for 108 young people (n = 3 missing). Most young people only had one referral for mental health support (n = 59, 66% of young people with referrals). However, the remaining 44% (n = 31) had two or three referrals made within the first year of entering care. To manage this and allow clearer reporting of referral information, we give descriptives of just one of the referrals made within their first year in care, rather than all referrals. To decide which referral to describe here, we pre-determined a hierarchy for selecting the referral. First, where there were multiple referrals, we report information from the last referral made whereby the young person accessed support. This was done because in cases of multiple referrals, earlier referrals were usually for triaging purposes, rather than support delivery. The only exception to this rule was where latter referrals were for carer-focussed interventions, and the former was young-person focussed (e.g., psychoeducation, play-based therapy). In which case, the young person focussed referral is selected in the hierarchy. This was done to allow for description of the support offered to the young people themselves, where applicable.

**Managing multiple treatment recommendations**

In a small number of instances, recommendations were made for more than one treatment within the referral or assessment letters (n = 5). Where this occurred, in order to explore the psychological support on offer to the young people specifically, interventions which were young person focussed (e.g., psychoeducation, play-based therapy), as opposed to carer-focussed, or psychopharmacology were taken as primary treatment recommendation and are reported in this research.

**Appendix S2.**

| Table 1. Discordant RCI and CCT cases | | | | |
| --- | --- | --- | --- | --- |
| **Year 1 to Year 2** | | | | |
|  |  | **Crossing Clinical Threshold** | | |
| **Reliable Change Index** |  | No Change | Borderline range | Normal range |
|  | No Change | 32 (23%) | 8 (10%) | 0 |
|  | Reliably Improved | 10 (12%) | 9 (11%) | 23 (28%) |
| **Year 1 to Year 3** | | | | |
|  |  | **Crossing Clinical Threshold** | | |
| **Reliable Change Index** |  | No Change | Borderline range | Normal range |
|  | No Change | 28 (39%) | 3 (4%) | 0 |
|  | Reliably Improved | 10 (14%) | 11 (15%) | 20 (28%) |
